# Current gynaecological management of women and girls with bleeding disorders in the United Kingdom: A UKHCDO haemophilia treatment centre survey and evaluation of real‐world clinical practice for the *British Journal of Haematology*

**DOI:** 10.1111/bjh.70295

**Published:** 2025-12-19

**Authors:** Laura Knox, Imogen Swart‐Rimmer, Naim Rahimi, Callum Harris, Lugain Abdalla, Gary Benson, Clare Brown, Helen Campbell, Ana Carvalhosa, Justin T. Clarke, Sarah Garside, Claire Lentaigne, Jayna Mistry, Priyanka Raheja, Cora Warren, Rezan Abdul‐Kadir, Gill Lowe, Nicola Curry

**Affiliations:** ^1^ Department of Haematology Glasgow Royal Infirmary Glasgow UK; ^2^ Department of Haematology University Hospitals Birmingham UK; ^3^ Oxford Haemophilia & Thrombosis Centre Oxford University Hospitals NHS Foundation Trust Oxford UK; ^4^ Sheffield Haemophilia Comprehensive Care Centre Sheffield NHS Foundation Trust Sheffield UK; ^5^ Northern Ireland Haemophilia Comprehensive Care Centre & Thrombosis Unit Belfast City Hospital Belfast UK; ^6^ Department of Haematology The Christie NHS Foundation Trust Manchester UK; ^7^ Patient Partner of the UKHCDO Girls and Women's Bleeding Disorders Group Manchester UK; ^8^ Southampton Haemophilia Comprehensive Care Centre University Hospital Southampton NHS Foundation Trust Southampton UK; ^9^ Department of O&G University Hospitals Birmingham UK; ^10^ Leeds Children's Hospital and The North and West Yorkshire Haemophilia Network Leeds UK; ^11^ Department of Haematology University Hospitals Plymouth NHS Trust Plymouth UK; ^12^ Adult Comprehensive Care Haemophilia Unit Queen Elizabeth Hospital Edgbaston Birmingham UK; ^13^ Department of Cardiovascular Sciences University of Birmingham Edgbaston UK; ^14^ The Royal London Hospital Haemophilia Comprehensive Care Centre The Royal London Hospital London UK; ^15^ Cardiff Haemophilia Centre and Bleeding Disorders Network Wales University Hospital of Wales Cardiff UK; ^16^ Department of O&G and Katharine Dormandy Haemophilia and Thrombosis Centre The Royal Free NHS Trust and Institute for Women's Health, UCL London UK; ^17^ Radcliffe Department of Medicine Oxford University Oxford UK

**Keywords:** gynaecological care, heavy menstrual bleeding, inherited bleeding disorders

## Abstract

Girls and women with bleeding disorders (GWBD) comprise more than half of all registered patients with bleeding disorders in the UK National Haemophilia Database. The gynaecological care of GWBD, until recently, has not been prioritised despite high health burdens, where four of every five patients experience heavy menstrual bleeding (HMB). We report the results of a national survey exploring gynaecological health‐care services offered across haemophilia centres in the United Kingdom, with a focus on HMB. We combine these results with a retrospective cohort analysis of individual patient care records, across a 3‐year period. Of 65 haemophilia centres, 41 responded, covering 90% of the UK GWBD population. Six hundred and ninety‐seven individual patient care records were included, from 13 centres. Our results show that immediate clinical care offered to GWBD experiencing HMB is adequate, despite infrastructure deficiencies (such as lack of joint‐gynaecology input and few centres having named clinical leads for GWBD). We recommend several areas for immediate prioritisation within haemophilia centres which will improve the equity of care for GWBD. These include direct access to gynaecological services; universal testing of iron status; and more broadly, a shift towards clinical practices that recognise and address the impact HMB has on patients' psycho‐social, sexual and overall quality of life.

## INTRODUCTION

The care of girls and women with inherited bleeding disorders (GWBD), which includes carriers of haemophilia and conditions such as von Willebrand disease (VWD), has historically been less intensive than the management of men and boys with haemophilia.^1^, ^2^ Heavy menstrual bleeding (HMB) is the most common symptom experienced by GWBD, affecting more than 80% of individuals.^3^, ^4^, ^5^ It may be the only symptom experienced during adolescence and, across all those affected with HMB, the duration and extent of bleeding are greater for GWBD than for those without a bleeding condition.^6^ HMB confers significant health, emotional and societal burdens, having the potential to impact all aspects of an individual's life.^7^ This includes both the physical effects—often relating to iron depletion—as well as the less appreciated effects on quality of life.^8^, ^9^, ^10^

Further to the challenges that an individual with HMB faces personally are the compounding issues that relate to the openness with which discussions around menstrual health are undertaken by clinicians^11^ and a reluctance by GWBD to seek advice regarding menstrual concerns, worsened both by familial normalisation of what constitutes abnormal/heavy menstrual blood loss^12^ and the commonly reported feelings of shame when discussing menses.^13^ To address these issues, a report from the European Haemophilia Consortium (EHC) and the European Association for Haemophilia and Allied Disorders (EAHAD) set out recommendations to prioritise the needs of GWBD.^14^ Added to this, the UK Haemophilia Centre Director's Organisation (UKHCDO) published their recommendations on the gynaecological care of GWBD.^15^, ^16^

In July 2025, the UK Haemophilia Society presented evidence to Parliament summarising their findings from extensive patient and public involvement, highlighting the gaps and inequalities in care for GWBD.^17^ The most recent UKHCDO annual figures (2023–2024) reported that there were 40 565 patients registered in the United Kingdom with bleeding conditions, of which 21 942 (54%) are GWBD.^18^ Notably, despite GWBD comprising more than half the patient population, it is only the most recent UK national specification for the management of patients with inherited bleeding disorders where recommendations for gynaecological care of GWBD are described.^19^ This study aims to describe contemporary practices of care, relating to the gynaecological health of GWBD, both at a centre‐level and patient‐level across the United Kingdom, to highlight variation in practices and provide a benchmark from which clinical services can develop.

## METHODS AND MATERIALS

We conducted a project with two arms: (1) a national survey of haemophilia centre‐specific clinical standards of practice and (2) a patient‐specific analysis of GWBD reviewed in clinic appointments across a 36‐month period.

### Clinical centre survey

We sent a pre‐piloted survey, using Survey Monkey, to all haemophilia centres in the United Kingdom (e.g. England, Wales, Scotland and Northern Ireland). The full survey can be found in Table S1.

The initial survey request was sent from the UKHCDO management team to the Haemophilia Centre Directors via email, and a reminder was sent 4 weeks later. Following this, personal direct emails were sent to the directors of those centres where no response had been received (both large comprehensive care centres [CCC] and smaller haemophilia centres [HC]).

### Individual clinic management case study

We used a cross‐sectional approach to evaluate current practice at the patient level for newly registered GWBD. A pilot was conducted at four centres using a prespecified audit tool (Table S2) with focused data collection based on recommendations set out in the UKHCDO Guideline.^15^ The study was extended to the remaining UK CCC and HC via an email invitation from the UKHCDO management team. Local audit approval was sought at each site.

### Individual case study, whole cohort

Individuals were included via a two‐step process: all females aged 10–55 years, who were menstruating, and registered between 1 January 2021 and 31 December 2024 were eligible. Case notes were screened for reports of HMB, either at diagnosis or during any documented clinical contact from the time of registration across the 36 months. Exclusions were patients diagnosed in the paediatric service or a different geographical region and transferred to a participating adult site to standardise results for each centre. Demographic data were extracted for all included individuals (age, diagnosis, bleeding assessment tool [BAT] score at diagnosis).

### HMB group

For those individuals experiencing HMB, further data were collected. Fully anonymised data were sent from participating sites to the study team for analysis. Data from the largest centres were directly compared to further evaluate clinical variability.

### Data analysis

Statistical analyses were performed on GraphPad Prism (v10.3.1) or SPSS (v31). Normality was assessed using visual histogram assessment and the Shapiro–Wilk test. Results were represented by mean ± SD/median ± interquartile range (IQR), comparisons made using *t*‐tests or Mann–Whitney, as appropriate. ANOVA was chosen to compare results across the six larger centres (Table 3) with binomial logistic regression to further interrogate results. Significance was set at *p* < 0.05.

## RESULTS

### Survey

Online questionnaires were distributed to 65 centres, including 29 CCC and 36 HC. Of the CCC, seven treated adults alone, seven children (age 0–18 years) alone and 15 treated patients across their lifespan. Responses were received from all (*n* = 29, 100%) CCC and 33% (*n* = 12) of the HCs. Ten of the included HCs treated adults and children, two adults only. The 41 centres covered 89.8% of the UK patient population with an inherited bleeding disorder (male and female) and specifically 92.3% of registered GWBD.

Of the 41 centres, many (*n* = 23, 56%) did not have a dedicated clinical lead (haemophilia specialist nurse or doctor) for GWBD and most did not have access to joint gynaecology–haematology clinics (*n* = 32, 78%). Despite this, two‐thirds of respondents had a named gynaecology link with whom patient care could be directly discussed. Only one in three (14/41) centres had a local guideline that offered recommendations on the gynaecological care of GWBD. Of these 14 centres, all 14 detailed how to manage acute onset HMB; 13/14 covered longer term management; 10/14 covered education around menstrual health and management of the menarche; and 11/14 covered the management of gynaecological surgery/procedures. Of 41 centres, 39 reported that formal surgical haemostatic management plans were provided.

Across the 41 respondents, 28 centres routinely reviewed all GWBD in their clinics (68%), 10 centres saw patients only if they had low factor levels and two offered patient‐initiated follow‐up. These percentages did not differ between CCC and HC. Just over half of all respondents (*n* = 22, 55%) provided a patient information leaflet (PIL) on menstrual health, with varying responses as to whether these were centre‐specific, age‐specific or accessed via external services such as the Haemophilia Society (Table 1). All but one centre proactively asked patients about menstrual health during a clinic appointment, and the remaining centre discussed menstruation if the patient asked about their periods.

**TABLE 1:** Descriptive data covered by respondents during the national survey.

	CCC	HC
Number of GWBD	17 031	3229
Number of centres	29/29	12/36
Clinical lead for GWBD	12/29	4/12
Named gynaecology link	20/29	5/12
Joint haematology–gynaecology clinics	8/29	1/12
Centre‐specific gynaecology guidelines	13/29	1/12
PIL covering menstrual health	20/29	4/12
Menarche plan as standard (applicable to fewer CCC/HC)	5/24	1/7
Ongoing review of patients until HMB resolved	28/29	11/12
Gynaecological surgical haemostasis plans written	28/29	12/12

Abbreviations: CCC, comprehensive care centre; GWBD, girls and women with bleeding disorders; HC, haemophilia treatment centre; HMB, heavy menstrual bleeding; PIL, patient information leaflet.

#### Age‐specific stages of life

73% (*n* = 30) centres provide care for GWBD in the pre‐menarche stage and all offer education to patients about HMB, with 94% also educating the family/carers. Notably, fewer centres educated the patient and/or family about what constitutes a normal menstrual cycle (70% and 61.3% respectively). One in four centres discussed how to manage menarche with the patient and/or checked iron stores pre‐menarche. Half of centres checked haemoglobin at this life stage.

At the time of menarche, one in five centres (20%) uses a written menarche plan as standard for all GWBD, which includes haemophilia clinical team contacts for the patient, in case of need. 45% of centres routinely ask about menarche in clinic but do not actively offer an individualised haemostasis plan. One centre confirmed that the GP manages the menarche.

**FIGURE 1:**
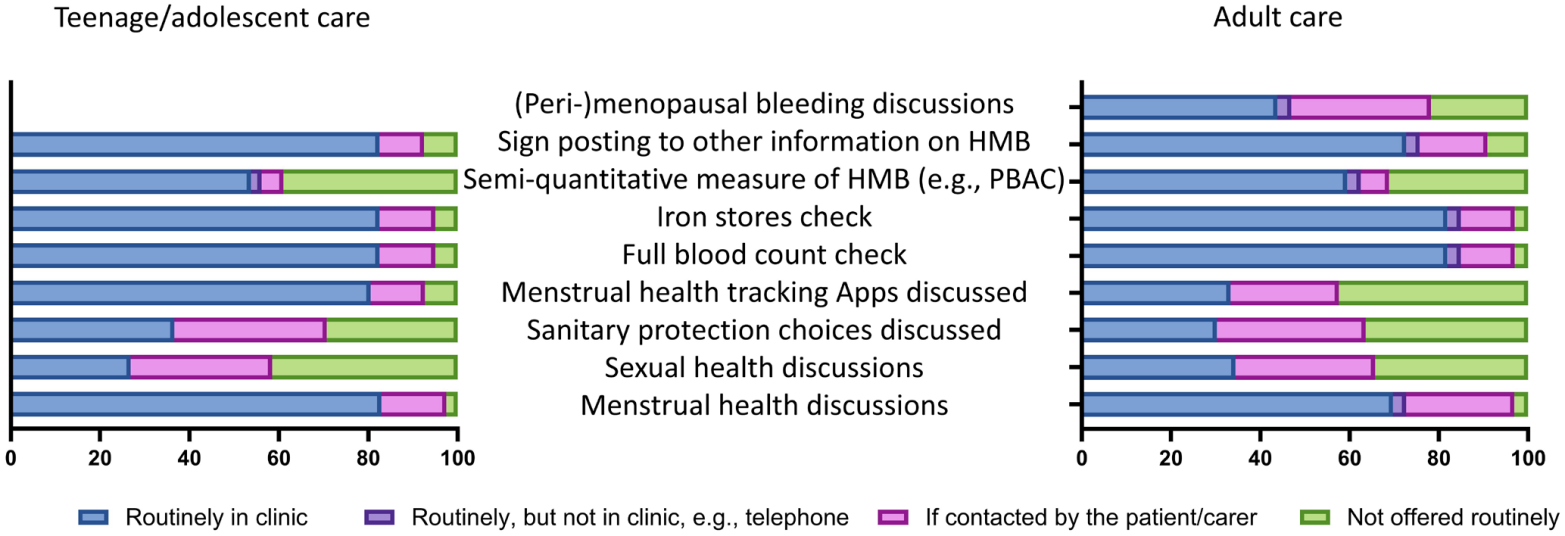
Day‐to‐day management of GWBD and their gynaecological health in the clinic setting, teenage versus adult populations. Forty‐one centres provided answers about adolescent care, since all centres cover this ‘cross‐over’ age range. Thirty‐three centres responded about adult care only. These histograms show the overall percentage of responses to each question, according to whether the response was fully affirmative (dark blue); partially affirmative (purple); conditionally affirmative (pink); or refuted (green). Data relating to teenage/adolescent care are shown in the left, and for adult care, it is shown in the right. One additional question was asked regarding the care of adults—relating to the (peri‐)menopausal life stage. HMB, heavy menstrual bleeding; PBAC, pictorial bleeding assessment chart.

Post‐menarche the care offered to young adults/teenagers does not differ from adult care (Figure 1). The fewest positive responses across both age groups related to discussions around the impact of a bleeding disorder on sexual health; the breadth of sanitary protection choices available and how to monitor menstrual flow to flag times of concern. Peri‐menopausal menstrual health was also an area infrequently discussed.

HMB was monitored by a variety of methods at 32 centres (78%). In 26 centres, a mix of the ISTH BAT^20^ and/or patient discussions were used, and in six centres, the PBAC was used. No centre used a questionnaire, such as the SAMANTA, which has been designed for menstrual health.^21^ 95% of centres follow a GWBD experiencing HMB until the symptoms have resolved, and most of these centres provide that care directly although, in six cases, the joint haematology–gynaecology clinic offers this service. Two centres direct the individuals to the GP for routine HMB care.

Biomarker measurements were evaluated commonly: most centres routinely offering a full blood count and iron status check (*n* = 40, 98%). (Iron studies were defined as a measurement which included some or all of ferritin, transferrin saturations, total iron binding capacity and serum iron levels.) 63% (*n* = 26) reported that they would repeat iron studies in a previously iron deplete/anaemic patient. In more routine settings, outside HMB, 49% (*n* = 20) centres would check iron status at the time of diagnosis of an inherited bleeding condition; 29% would check at every clinic for menstruating females; 41% during pregnancy and 34% after delivery.

**FIGURE 2:**
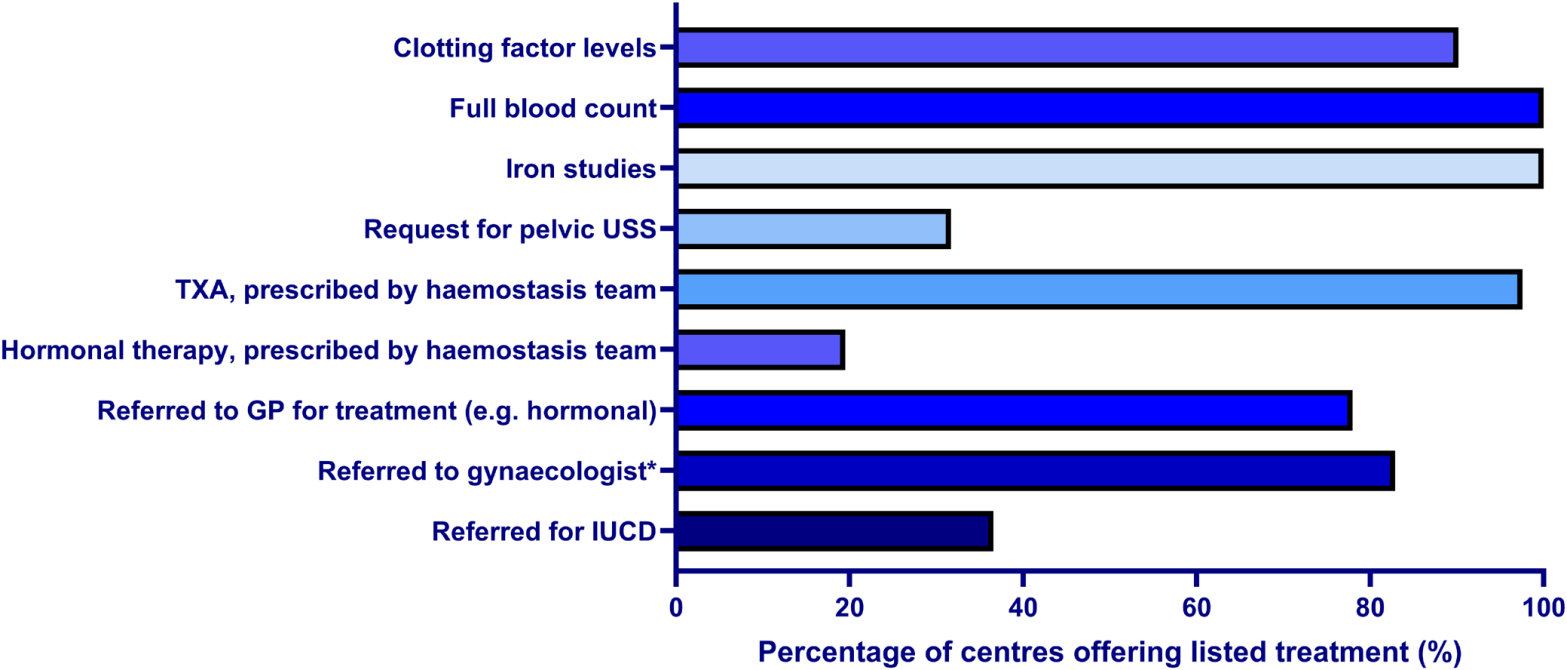
Immediate management by haemophilia treating teams when a patient presents with HMB, not requiring hospital admission. The histogram shows percentages of the total number of respondents (*n* = 41) answering that their centre's standard practice involves each listed management strategy. *‘referred to gynaecologist’ included 25 centres specifying referral to gynaecology clinic and an additional nine centres referring to a joint haematology–gynaecology clinic. GP, general practitioner; IUCD, intrauterine contraceptive device; TXA, tranexamic acid; USS, ultrasound scan.

Clinicians reported that they would refer a patient to gynaecology or their joint haematology–gynaecology clinic: 11/41 routinely referred when HMB is diagnosed (Figure 2); 24/41 referred only if first‐line therapy failed, for example, when HMB persisted after the use of a single agent tranexamic acid or hormonal therapy; five centres referred for patients to discuss insertion of a Mirena coil or other intrauterine system (IUS); five centres referred when HMB was impacting quality of life; five referred to exclude/investigate structural causes; and seven centres referred for the management of ongoing iron depletion.

### Individual case study

**FIGURE 3:**
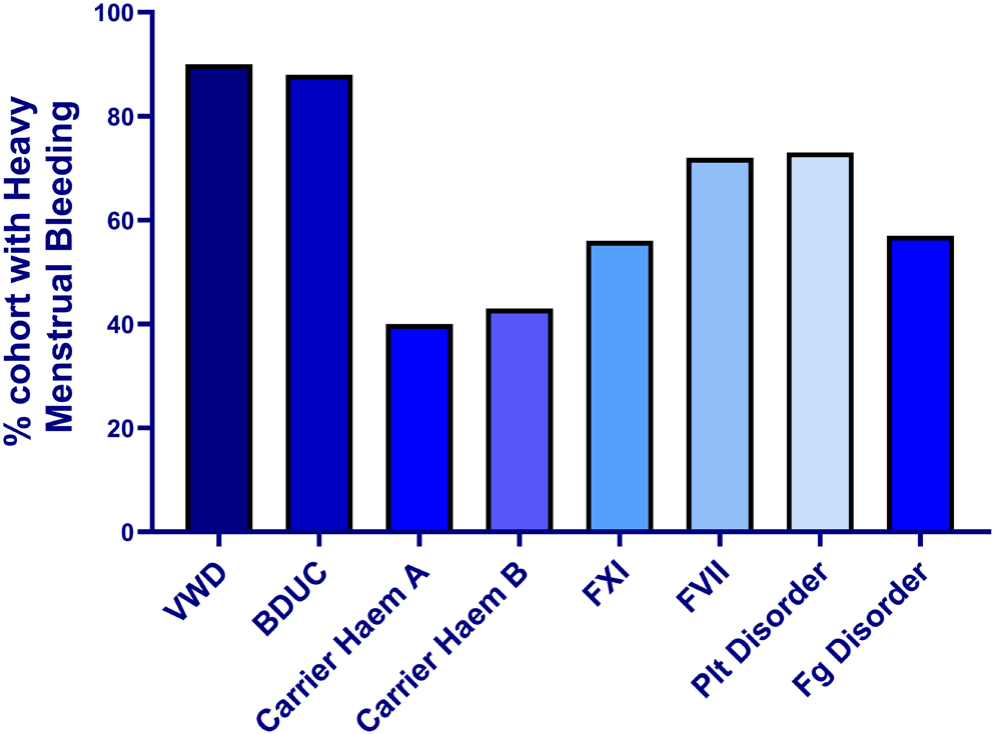
Percentage of GWBD experiencing HMB, according to their registered inherited bleeding disorder type. BDUC, bleeding disorder of unknown cause; F, factor; Fg, fibrinogen; VWD, von Willebrand disease.

Seven hundred and twenty‐seven patients were identified at 13 centres (11 CCC, 2 HC) with a newly registered bleeding disorder between 1 January 2021 and 31 December 2024. Thirty individuals had moved centre and were deemed ineligible. In total, 697 patients were included. The mean age at diagnosis was 28.9 years (SD: 9.7), range: 8–55 years. Two hundred and twenty‐eight individuals (32.7%) did not experience HMB, and 469 (67.3%) did experience HMB. There was no difference between those without or with HMB; mean—29.1 years (SD 9.9), range 8–50 years, versus 28.8 years (SD 9.6), range 8–55 years, respectively, *p* = 0.73 (Table 2). As previously described, we found a variable likelihood of individuals experiencing HMB, according to their bleeding disorder diagnosis. Our data confirm a preponderance of HMB in those with VWD, bleeding disorder of unknown cause (BDUC), platelet disorders and FVII deficiency (Figure 3).

**TABLE 2:** Characteristics of whole cohort and according to the presence or absence of HMB.

	Whole cohort (*n* = 697)	Non‐HMB (*n* = 228)	HMB (*n* = 469)
ISTH BAT score, median, (IQR)	5 (IQR 6)	1 (0–3)	7 (4–9)
BAT score documented, *n* (%)	376 (54)	105 (46)	271 (58)
Age at diagnosis, mean, (SD)	28.9 (9.7)	29.1 (9.9)	28.8 (9.6)
*Type of IBD, as registered by the National Haemophilia Database, n (%)*			
BDUC	122 (17.4)	15 (6.6)	107 (23)
VWD^a^	78 (11)	8 (3.5)	70 (15)
Low VWF	11 (1.6)	4 (1.8)	7 (2)
Haemophilia A Carrier	128 (18.4)	77 (34.0)	51 (11)
Haemophilia B Carrier	21 (3)	12 (5.0)	9 (2)
FVII deficiency	61 (8.7)	17 (7.5)	44 (9)
FXI deficiency	80 (11.5)	35 (15.3)	45 (10)
Platelet dysfunction^b^	107 (15.3)	28 (12.3)	79 (16.8)
Disorders of fibrinogen	46 (9.8)	20 (8.7)	26 (6)
Acquired Haemophilia or VWD	3 (<1)	3 (1.3)	0 (0)
Other single factor deficiencies^c^	18 (2.6)	5 (2.2)	13 (3)
Other/combined^d^	22 (3.1)	4 (1.8)	18 (3.8)

Abbreviations: BAT, bleeding assessment tool; BDUC, bleeding disorder of unknown cause; F, factor; HMB, heavy menstrual bleeding; ISTH, International Society for Thrombosis and Haemostasis; VWD, von Willebrand Disease; VWF, von Willebrand Factor.

^a^ Not all data regarding subtype of VWD were available for all participants; therefore, further classification is not given.

^b^ Including (and not limited to) Glanzmann's thrombocythaemia, Bernard–Soulier syndrome, GP1BB‐associated mild macrothrombocytopenia, GATA1‐associated thrombocytopenia, MYH9 thrombocytopenia.

^c^ Including (and not limited to) Factor V, Factor X and Factor XII deficiency.

^d^ Including Ehlers–Danlos syndrome, other hypermobility syndromes, combined factor deficiencies and combined pathology.

#### HMB group

91% of the HMB cohort (*n* = 428/469) had a documented full blood count. Only 64.7% had a documented ferritin (*n* = 302). Two‐fifths of the overall HMB cohort (*n* = 180), or more pertinently, 59.2% of those tested with a ferritin, had confirmed iron deficiency (e.g. ferritin <30 μg/L), 144 of whom were on iron replacement (either IV or PO). Thirty‐six iron‐deficient patients (20%) did not have evidence of iron being prescribed. Just over one‐third (36.7%) had evidence of a pelvic US having been requested—although in one‐third of this group (*n* = 55/172), there was no documentation to confirm whether this had occurred. 12% were planned for, or had been investigated with, a hysteroscopic procedure.

UKHCDO recommendations are that a menstrual plan be in place for all GWBD who menstruate. 65% (*n* = 166) did have one. Furthermore, 75% of the HMB cohort had documentation outlining the use of tranexamic acid (TXA) for treatment. The UKHCDO guideline recommends TXA as a first‐line treatment for women trying to conceive or when hormonal therapies are not acceptable. TXA is also recommended in combination with hormonal therapies to improve treatment efficacy. Oral hormonal therapy was discussed with 58% (*n* = 258/469); discussions around IUS were documented for 29% of individuals. Only one‐third of patients had active gynaecological involvement. One in five individuals was directed to educational patient information on menstrual care, either using centre‐specific leaflets or leaflets from the Haemophilia Society. Most had no documented discussion around possible surgical management options (70%, *n* = 308). Moreover, importantly, 9% (*n* = 42) of individuals had an unscheduled hospital attendance (e.g. visit to the emergency department) for HMB symptoms.

Documentation detailing the social impact of HMB was poor: 50 individuals (10%) were noted to have absences from school or work, and in two‐thirds of notes, there was no documentation as to whether this impact of HMB had been explored. 20% (*n* = 93) of the group reported fatigue, but again half (*n* = 206) of individuals were not asked about this symptom.

### Centre‐specific variation

Finally, we explored individual centre differences, with a focus on HMB prevalence and evaluation of iron deficiency. We compared six large centres (e.g. returning >65 patient entries), Table 3. Prevalence of HMB varied (53–73%). Logistic regression analysis confirmed that the bleeding condition diagnosis conferred the strongest influence on this difference (*p* < 0.001) rather than a difference between centres. Clinical practices for checking iron status were highly variable, with between one‐quarter and 100% of individuals having their iron levels checked. For those with a ferritin showing iron depletion/deficiency, variability in treatment was evident, and in two centres, fewer than two in three iron‐deficient patients received therapy.

**TABLE 3:** Comparison of six larger centres.

	Centre 1 (*n* = 106)	Centre 2 (*n* = 106)	Centre 3 (*n* = 104)	Centre 4 (*n* = 117) cant	Centre 5 (*n* = 68)	Centre 6 (*n* = 95)
*Data for whole cohort from each centre*						
Mean age at diagnosis, (SD)	30.6 (7.5)	31.8 (8.0)	27.4 (9.9)	25.6 (11.9)	31.8 (13.7)	31.5 (8.7)
BAT recorded, *n* (%)	88 (83.0)	105 (99.1)	15 (14.4)	33 (28.2)	65 (95.6)	10 (10.5)
*Centre prevalence of HMB*						
HMB, *n* (%) of centre cohort	59 (55.7)	78 (73.6)	70 (67.3)	79 (67.5)	36 (52.9)	60 (63.2)
Documented to not to have HMB, *n* (%)	38 (35.8)	28 (26.4)	34 (32.7)	37 (31.6)	29 (42.6)	32 (33.7)
No documentation about HMB, *n* (%)	9 (8.5)	0 (0)	0 (0)	1 (0.9)	3 (4.4)	3 (3.1)
*Data for individuals with documented HMB*, *n* (%)						
Ferritin checked	38 (64.4)	48 (61.5)	40 (57.1)	21 (26.6)	36 (100)	59 (98.3)
Iron deficiency confirmed^a^	17 (44.7)	25 (52.1)	22 (55.0)	12 (57.1)	23 (63.9)	12 (20.3)
IDA treated^b^	5 (29.4)	24 (96.0)	22 (100)	7 (58.3)	18 (78.3)	8 (75.0)
US performed	21 (35.6)	23 (29.5)	23 (32.9)	17 (14.5)	21 (58.3)	20 (33.3)
Active gynaecology input	14 (23.7)	23 (29.5)	12 (17.1)	27 (34.2)	17 (47.2)	20 (33.3)
HMB treatment plan in place	19 (32.2)	29 (37.2)	59 (84.3)	42 (53.2)	28 (77.8)	40 (66.7)
*Treatments discussed and/or offered to those with HMB*, *n* (%)						
Tranexamic acid	27 (45.8)	63 (80.7)	60 (85.7)	66 (83.5)	29 (80.6)	39 (65.0)
Hormonal therapy	26 (44.1)	55 (70.5)	29 (41.4)	35 (44.3)	21 (58.3)	35 (58.3)
Progesterone IUCD	9 (15.2)	33 (42.3)	17 (24.2)	17 (21.5)	5 (13.9)	24 (40.0)
Surgery	7 (11.9)	15 (19.2)	3 (4.3)	10 (12.7)	7 (19.4)	13 (21.7)

Abbreviations: BAT, bleeding assessment tool; HMB, heavy menstrual bleeding; IDA, iron deficiency anaemia; IUCD, intrauterine contraceptive device; US, ultrasound.

^a^ In those having had a ferritin taken.

^b^ In those with a ferritin <30.

## DISCUSSION

Equity of access to, and the provision of, high quality care is a central tenet of good healthcare. However, the impact of HMB despite its universality, and the management of gynaecological health more broadly for GWBD, has not been prioritised. In the last few years, these health inequalities have become more prominent.^22^ This descriptive analysis was conducted in recognition of these inequalities and to better understand contemporary clinical care offered across the United Kingdom to highlight areas for harmonisation and improvement.

The number of respondents to our survey was high, with all 29 CCC responding, covering more than 90% of registered patients. As such, our data are highly representative of the care offered across the United Kingdom. The results confirm that just over half of the centres lack a clinical lead focused on women's health, only one‐third of centres have local guidance to direct clinical care, and only one in five centres have joint clinics. These results are less favourable than those from a European survey, where twice as many centres offered joint gynaecology clinics and had local management algorithms for all aspects of gynaecological care.^23^ These differences may, in part, reflect inclusion bias in the European survey (40% response rate), whereas our data included all CCC, or it may more broadly reflect country‐specific differences.

There was evidence of good practice in some aspects of care for GWBD. Education around HMB was universally offered at the pre‐menarche life stage, and similarly, all centres discussed HMB across all age groups. Added to this, most centres offered a standard approach to HMB diagnosis—with blood tests, an anti‐fibrinolytic medication, and then—generally—referral to start hormonal control methods. The individual clinical case study confirmed this common approach. Interestingly, within the near 700‐strong patient number evaluated, only two‐thirds were documented to have HMB—a figure that is considerably lower than other publications.^3^, ^4^, ^5^ This may represent poor clinical capture of HMB and/or may reflect that many publications report survey results, which can suffer reporting bias.

There were, however, large variations in the longer term management of HMB. Notably, few patients were managed with active gynaecological input (32%). Greater communication and joint working, with cross‐fertilisation of best practices between specialities, would improve the confidence of haematologists to manage HMB and could have a rapid beneficial impact on patient care. Nevertheless, there are barriers to joint working, including consultant time, access to funding to set up services and a variability of prioritisation between hospitals which would need to be addressed to support adoption of these clinics.^24^, ^25^

Our patient partner stressed the importance of joint haematology–gynaecology care and that excessive bleeding during perimenopause and bleeding relating to sex should be proactively addressed in consultations. Furthermore, clinicians should go further to support requests by patients for reasonable adjustments at work for HMB to facilitate ongoing employment.

In resource‐rich countries, HMB is one of the most common causes of iron deficiency.^26^ Our data highlight opportunities to improve screening for iron depletion in our at‐risk patients. Iron loss is associated with significant health burden, further compounded by societal and economic costs. The impact of absenteeism annually in the United Kingdom from HMB is £4.7 billion.^27^ These data highlight the urgent need for routine clinical practices, around detection and monitoring of iron deficiency, to change.^28^ The authors advocate annual monitoring of iron status for all GWBD who are menstruating.

There are limitations to our data. Although 90% of the registered UK GWBD were included in the centre‐specific survey, we were unable to include all HCs. Survey data can be skewed, according to the individual and the patient mix of the centre where the questionnaire is completed. Notably, only 11% of the audit cohort were diagnosed with VWD, which is unexpectedly low, given that in the United Kingdom, each year, approximately 25% of newly registered females have VWD. Our clinical audit data reflected the answers provided in the survey, which was reassuring. We did not collect ethnicity or socioeconomic data (including the impact of period poverty on patients), which would have been of particular interest given several inherited bleeding disorders have a higher prevalence in ethnic minority populations. We did not ask if written material was provided in patients' first spoken language. ISTH BAT score reporting^29^ varied significantly between centres, making the results for this criterion less broadly applicable.

## CONCLUSION

Our comprehensive national survey shows that clinical care offered to GWBD experiencing HMB is adequate, with much room for improvement. Areas for immediate prioritisation include greater access to gynaecological services; universal, routine testing of iron status; and more broadly, a shift towards clinical practices that recognise and address the impact HMB has on patients' psycho‐social, sexual and quality of life.

## AUTHOR CONTRIBUTIONS

Laura Knox, Imogen Swart‐Rimmer, Naim Rahimi, Callum Harris, and Lugain Abdalla designed the audit of practice, piloted and completed the audit; Gill Lowe and Nicola Curry supported the audit project, reviewed and analysed audit data. Gary Benson, Ana Carvalhosa, Justin T. Clarke, Sarah Garside, Claire Lentaigne, Priyanka Raheja, Cora Warren, Rezan Abdul‐Kadir, Gill Lowe, and Nicola Curry designed the survey of clinical centres. Nicola Curry collated and analysed the data; Nicola Curry and Laura Knox wrote the first draft of the manuscript. All authors reviewed and updated the manuscript.

## FUNDING INFORMATION

The author(s) received no financial support for the research, authorship and/or publication of this article.

## CONFLICT OF INTEREST STATEMENT

The authors declare no competing financial interests in relation to this manuscript.

## ETHICS STATEMENT

Formal ethics were not required for the national survey, and all participating sites followed local approval practices prior to undertaking the audit.

## Supporting information


Table S1.

Table S2.

## Data Availability

All data are available on request from the corresponding author.
