# Supplementary material for: Current gynaecological management of women and girls with bleeding disorders in the United Kingdom: A UKHCDO haemophilia treatment centre survey and evaluation of real‐world clinical practice for the British Journal of Haematology
Source: Br J Haematol. 2025 Dec 19;208(2):661–9. doi: 10.1111/bjh.70295 (PMC12916183; doi:10.1111/bjh.70295)
Supplement: Supplementary file 1 — Table S1. [file BJH-208-661-s001.docx]

**Supplementary Table 1.**

**Data Collection Form GWBD. Haemophilia Centres.**

1.What is the name of your haemophilia centre?

2.Does your centre look after adults or children?

- Adults only

- Children and young adults (e.g. 0-18 years) only

- All ages

3.How many patients with inherited bleeding disorders are registered at your centre?

4.How many girls and women with inherited bleeding disorders (GWBD) are registered at your centre?

5.Do you have a haemophilia centre clinical lead for GWBD?

Yes / No

6.Do you have a named gynaecologist/gynaecology team with which your service links?

Yes / No

7.Does your service run joint gynaecology/haematology clinics?

Yes / No

8.Do you have a centre-specific SOP about how to manage the gynaecological care of GWBD?
(e.g. an SOP which might cover all/part of the following: care of the menarche, menstrual cycles,(peri-) -menopause, surgery/procedures).

Yes / No

9.In your SOP for gynaecological care do you cover the following topics?

- provision of education re. menstrual health

- management of menarche

-management of acute HMB

-longer term management of HMB

-management of gynaecological procedure or surgery

10.In your practice, does your centre routinely offer clinic appointments to all GWBD?
-Yes, we routinely see all GWBD

-We routinely see all GWBD during their reproductive lifetime

-No

-We see only those with low factor levels

-We offer PIFU (patient initiated follow up)

11.Does your centre have a patient information leaflet (PIL) that can be given to patients covering menstrual health

If you look after girls and young adults, do you have different PIL according to ages

-Yes - one PIL for our adult population specifically

-Yes - one PIL for our paediatric population specifically

-Yes - one PIL that we give for all patients, irrespective of age

-Multiple PILs according to age of the patient

-No

12.In your centre's practice, do you provide surgical plans for gynaecological operations?

-Yes, we write formal haemostatic gynae plans

-No

13.Do you look after girls in the pre-menarche stage?

Yes / No

14.In the pre-menarche stage, which of the following is offered to your patient and her family/carer/guardian?

In these sections: 'iron studies' means ferritin as a minimum. In some centres this term may also include TIBC/iron/transferrin. Please tick all options that apply to your routine practice.

-education of the patient about normal menses

-education of the family/carer/guardian about normal menses (as directed by age of patient)

-education of the patient about the potential for HMB (age appropriate)

-education of the family/carer/guardian about the potential for HMB (as directed by age of patient)

-PIL on menstrual health

-a menarche plan is discussed and agreed

-iron studies are checked pre-menarche

-haemoglobin is checked pre-menarche

15.Do you look after GWBD during their menarche?

Yes / No

16.Does your centre use a 'menarche plan' as standard?

Yes / No

17.When a patient reaches menarche, how does your team routinely approach their care?

Please tick all that apply.

-We have an agreed menarche plan, and our patient/their carer contacts us routinely, whether or not there are concerns

-We have an agreed menarche plan and our patient/their carer contacts us if they need help and/or treatment due to HMB

-We ask the patient/their carer about menarche in clinic when they come for a routine appointment

-The care of HMB at the menarche is managed locally by the GP

18.Do you look after GWBD who are young adults / teenagers?

Yes / No

19.For your teenager / young adult, which of the following do you routinely provide regarding their gynaecological care?

Please tick all that apply.

-discussion around menstrual health – what is normal etc

-discussion around sexual health

-discussion of options for sanitary protection

-discussions around electronic menstrual health tracking apps

-FBC check

-iron studies check

-semi-quantitative measure of menstrual blood loss – eg PBAC, ISTH-Bat

-signposting to information: e.g. Haemophilia Society

20.Do you look after an adult population?

Yes / No

21.For your adult patient, which of the following do you routinely provide regarding their gynaecological care?

Please tick all that apply.

- discussion around menstrual health – what is normal etc

-discussion around sexual health

-discussion of options for sanitary protection

-discussions around electronic menstrual health tracking apps

-FBC check

-iron studies check

-semi-quantitative measure of menstrual blood loss – eg PBAC, ISTH-Bat

-signposting to information: e.g. Haemophilia Society

22.Is it your centre's practice to offer pre-conception counselling, including referral for PGD (where appropriate)?

Yes/No

23.For GWBD who are seen in clinic, is it your centre's practice to always ask patients about their menstrual health (where relevant)?

-Yes

-No

-Only if the patient asks about their periods

-Only if the patient has had problems with HMB in the past

24.When is it your standard practice to check iron studies in GWBD?
-At diagnosis

-To follow up on previous IDA/depletion

-At every clinic appointment for menstruating females

-During pregnancy

-After delivery

-We are led by the clinical picture

25.Do you regularly score for the presence of HMB in GWBD?
-Yes

-No

-Only when led by the clinical picture

26.When you score for the presence of HMB which tools are used at your centre?
-ISTH BAT section for menstrual health

-using patient discussion

-using a patient questionnaire - eg SAMANTA

-using the PBAC

27.When a GWBD presents with HMB, which of the following management is offered in the immediate setting (tick all that apply)

-clotting factor bloods

-FBC

-iron studies

-request for pelvic USS

-tranexamic acid (prescribed by the haemophilia team)

-hormonal therapy (prescribed by the haemophilia team)

-referred to GP for treatment (such as hormonal therapy)

-referred to gynaecologist

-referred for IUCD (e.g. Mirena)

-referred to haem-gynae clinic

28.When might you consider a referral to the gynaecology service?
 - freetext

29.Do you have access to a joint haematology/gynaecology service
Yes/No

30.When would you refer to your joint haem/gynae service?
-freetext

31.When HMB is diagnosed in your patients, how would your centre routinely monitor HMB response?

-It is our practice that the GP manages this

-We monitor in IBD clinic using PBAC

-We monitor in IBD clinic using qualitative information from our patients - eg reporting HMB improved/worsened

-Our joint haem/gynae service monitors HMB until it resolves

32.When a patient is being treated for HMB, is it your clinic's practice to follow up until HMB resolved?

Yes/No

**(Transcribed from online Microsoft Forms document, 21^st^ November 2025).**
