# Supplementary material for: Current gynaecological management of women and girls with bleeding disorders in the United Kingdom: A UKHCDO haemophilia treatment centre survey and evaluation of real‐world clinical practice for the British Journal of Haematology
Source: Br J Haematol. 2025 Dec 19;208(2):661–9. doi: 10.1111/bjh.70295 (PMC12916183; doi:10.1111/bjh.70295)
Supplement: Supplementary file 2 — Table S2. [file BJH-208-661-s002.docx]

Supplementary Table 2.

Audit Proforma Version 2.0

**Demographics**

1. Initials (Local only)
2. MRN (Local identified, Local only)
3. Paediatrics or Adult Patient
4. Age

**Diagnosis Details**

1. Diagnosis
2. Date of Diagnosis
3. Age at Diagnosis
4. ISTH BAT Score at Diagnosis
5. Who Referred (Primary or Secondary Care)

**HMB History**

1. Has HMB (Heavy Menstrual Bleeding) been diagnosed at any point during care?
2. Was HMB an issue at diagnosis?

**HMB Investigations**

1. Was FBC performed?
2. Was Ferritin performed?
3. Was TSH performed?
4. Was Transvaginal Ultrasound (TVUS) performed?
5. Was Hysteroscopy performed?

**Menstruation Plan**

1. Is there a documented menstruation plan?
2. Has TXA been discussed?
3. Has the Oral Contraceptive Pill (OCP) been discussed?
4. Has an Intrauterine Device (IUD) been discussed?
5. Who started the OCP?
6. Who fitted IUD?
7. Has joint care with Gynaecology been discussed or undertaken?
8. Has surgery been discussed?

**Impacts**

1. Has there been absence from School or Work?
2. Has there been unscheduled Hospital attendances?
3. How many unscheduled attendances?
4. Has the patient been signposted to patient information groups/services?
5. Is there evidence of iron deficiency?
6. Has iron deficiency been treated?
7. Is there any documentation of Fatigue?
